# Supplementary material for: Clone wars: asexual reproduction dominates in the invasive range of Tubastraea spp. (Anthozoa: Scleractinia) in the South-Atlantic Ocean
Source: PeerJ. 2017 Oct 5;5:e3873. doi: 10.7717/peerj.3873 (PMC5632532; doi:10.7717/peerj.3873)
Supplement: File S1 [file peerj-05-3873-s001.docx]

Supplemental File 1. Microsatellites accession numbers and sequences.

Tco1 – ACCESSION KY198738

acccggtcatgtaaggctgtacactgatctgtttaatcgttcgcnacgtaaaaantagggtgttttaactctacagatcagcttctgttttgttatattgtctaacctttattattttcaattatattacttttctctgcctcgaatagcgtttttgctaattcgcngggnaactgtacttaagttgatatgtctaataaagttttgtttgtttgttgttgttgttgttgtttgtagaatgttataataaggttcgtgaactcgcaaacgttgacgtcagagagtcgtcttaaacggcctcgtttccatgtaacgggtcttccaattaagaactcgtacagatagcagacaagagtgtctgaaatgaaatttgatgatgtctgtaagtacagactacgcctagtcaaagcaagtacccgtgct

Tco4 – ACCESSION KY198739

gtggagagtgaataagcttggggtgagtgaataaaagggggtaagtgaataagagggggnagtgaatattataaagagggggagtcgagtgaataagagggggntgaaaatgttagagacaagataccataaagttttgataatatcatcancatcatttattnattagccttttttttnaantaacagatttaacaagttacagcagnttgttaggcgaggagacctcaagaaaccatcaggc

Tco5 – ACCESSION KY198740

tcaggagccgattaatacctgattatttttctgtaagattaaggagactctacttattcatgaactcagacctacccttaacgataatgtctgattatttttgttattttaacgagtgtttngacataatgtagacaagataaaccattggaaaaataaaatattaatagcagcctttgttaaatcattgaaaaaagaacgttaacagcaatgcaatatcttaattattgggctcaatatataaccacagaagcttatcacgtatgagcttctgataaccataactcaaagttccagtttcagtgaaactggaaagaaagaangaaagaangaaatatttctttctttcttacgaatgaacgaatggacggacggacgaacggacgaacgaacgaacttgagcacattcactgcaca

Tco8 – ACCESSION KY198741

tgctgccgcgatacgagcatcgtttcatcacccccgagtattggcaagaggaagaggaggaggaggaggaggaagagtcgatggagtgaactgacacaaaacacgcgttttgttttacaagtggttgatttttattaaattttcaactcaacgttggattttacaataaagtgttcttttacaatttaaacgtttcttgactgcatgattttttcacaatcttctcngncngggtatnttttcccgncaggtngntgatcccncggancnttttttcgtccgctttccacttgtc

Tco9 – ACCESSION KY198742

ttgaccacgtactgccaagttcatctgccagtttttaatattatgatcgacagaactcgtaattttaaattaaaaactccaaacgttatcgtattaatttgttcctgactctttctgtgatagcgtcaccagcggccagtgtttattttcttgcttagagcctatctttcatatagaattgttatcttagtaatttactctgttattagttttctatttgaaaggcttgtcgagctgcattcaaaaccctatattaagtctcgtcaaactccaggcacattccacaatatatatatatatatatataggctgagccaaacagcggagctctctgaacaga

Tco29 – ACCESSION KY198743

gtgccctaggtccatggtttggacccggcccccatggagaacacataattatatagagaacacataataataataataataataataataataataataataataataataataataataatacaatgcactagaaatttataataacttaaaagacaatagactacgaaatgattaaggggtagtgacaaggaagcctatatagaagccgg

Tco30 – ACCESSION KY198744

gggaattcggatgcaattataacatacatacatacatacatacatactttattggctcgtccccatggggtttttcagagtcaatttaaaattacataactaagtacaagacaataaaagtaaaaagtaaaacttgtggattaacttaaaaagtcattaagaagtttttgcctaagtaagcgcttaaacaccatcacggaagggctaagtctgagcgagggttgcagctcattccacagag

Tco32 – ACCESSION KY198745

gcgtggtctggtcttttcattaaaaaagaatcagggtatcgttaccgttcaccagcttactgacttagtcattgctgtttggaacctctccggtaaaatataataataataataataataataataataataataataacgaacttattctttcgttttctctgcagttaatgatcagtttgagaagctatatcaaacactcgaaagagtgtttcattaggtatccaaacacctcaaagtgggt

Tco34 – ACCESSION KY198746

gcgcctactaccacacgaatggtttattattattattattattattattattattattattattattattattattattatggtacagttactttaattgcaaatggtttctcactctcaacctccgagtccaaaccatggacttagggatgcaaagaggatttctacttgatggaaagcacccgcctaagaaggtgcgctgtagaaagga

Tco36 – ACCESSION KY198747

gcaatgacaacagccagaacatgatgttaaacaaagataataataataataataataataataataataataataataattatcatcaagaacaacaaggacaagatatgcacacttatcgatgtagccattccctctgacaaaaacatctccaccaaagtctctgagaaaacatccaaatataaagacctagaacttgagattgcaagaatgtggcagacgaaa

Tco37 – ACCESSION KY198748

aaacattcgattcccactcggcaatcttgtaaatcagtttttatagttattttaatttttataaatgattgtaattaaaatactattagtgtaactgaagatttttaaccgagtttaaataaagatctactactactactactactactactactactactactactactactactactactactactactactactacggatgcggtatataatttgaacggaaatattagtggccgggt

Tco38 – ACCESSION KY198749

tttgagtttgagtttattgactccttagctacatacatacatacatacatacatactttattgaccacttccccaaaggggcttttcagtgccaattacaaagaaaaagctaaaaataaaatatatacaaattatttaaagttacaattacaaattattaggatatcattcctccctcttaaatttgtctaaaagcacccctctaagcttactcc
